# Resonance assignment of As-p18, a fatty acid binding protein secreted by developing larvae of the parasitic nematode *Ascaris suum*

**DOI:** 10.1007/s12104-012-9447-1

**Published:** 2012-12-06

**Authors:** Marina Ibáñez-Shimabukuro, M. Florencia Rey-Burusco, Alan Cooper, Malcolm W. Kennedy, Betina Córsico, Brian O. Smith

**Affiliations:** 1 Facultad de Ciencias Médicas, Instituto de Investigaciones Bioquímicas de La Plata, CONICET-UNLP, Calles 60 y 120, 1900 La Plata, Argentina; 2 School of Chemistry, University of Glasgow, Glasgow, G12 8QQ UK; 3 Institute of Molecular, Cell and Systems Biology, University of Glasgow, Glasgow, G12 8QQ UK; 4 Institute of Biodiversity, Animal Health and Comparative Medicine, University of Glasgow, Glasgow, G12 8QQ UK

**Keywords:** As-p18, Fatty acid binding protein, nemFABP, Nematode, Parasite, *Ascaris suum*

## Abstract

As-p18 is produced and secreted by larvae of the parasitic nematode *Ascaris suum* as they develop within their eggs. The protein is a member of the fatty acid binding protein (FABP) family found in a wide range of eukaryotes, but is distinctive in that it is secreted from the synthesizing cell and has predicted additional structural features not previously seen in other FABPs. As-p18 and similar proteins found only in nematodes have therefore been designated ‘nemFABPs’. Sequence-specific ^1^H, ^13^C and ^15^N resonance assignments were established for the 155 amino acid recombinant protein (18.3 kDa) in complex with oleic acid, using a series of three-dimensional triple-resonance heteronuclear NMR experiments. The secondary structure of As-p18 is predicted to be very similar to other FABPs, but the protein has extended loops that have not been observed in other FABPs whose structures have so far been solved.

## Biological context

As-p18 is a developmentally-regulated fatty acid binding protein found in the perivitelline fluid surrounding the infective third stage larva (L3) of the parasitic nematode *Ascaris suum*, which is closely related to the large intestinal roundworm of humans, *Ascaris lumbricoides*. Once fully developed, the L3 undergoes developmental arrest, persisting for several years until ingestion by the host, but little is known about the biochemical and physiological basis for such long term survival. One of the most abundant components in the fluid that surrounds the developing larva is the fatty acid binding protein, As-p18 (Mei et al. 1997). As-p18 displays significant sequence similarity to intracellular lipid binding proteins of vertebrates and other metazoan phyla, the best understood being the fatty acid binding proteins (FABPs) of humans. But, only in nematodes are they secreted from the synthesizing cell (Mei et al. 1997; Plenefisch et al. 2000). Modelling studies predicted that the protein conforms well to a ten β-stranded, two α-helix FABP, but that there are modifications to the binding site which could relate to ligand specificity. Moreover, unusual extra loops were predicted to extend from the surface of the protein that might be involved in previously unsuspected protein–protein or protein-membrane interactions related to the unusual location and function of the protein (Kennedy et al. 2000).

Phylogenetic analysis reveals that As-p18, together with potential homologues and paralogues found in *Caenorhabditis elegans*, comprise a distinct subclass (here named ‘nemFABPs’) within the wider FABP family that are possibly unique to nematodes (Plenefisch et al. 2000). The developmental control of As-p18’s expression, and the presence of a working secretory leader peptide encoded in the protein’s mRNA, also distinguish these nematode proteins from other FABPs. The unique features of As-p18 may reflect adaptation to a specific function within the egg related to the remarkable environmental resilience of the larva (Kennedy and Harnett 2001). Importantly, the perivitelline fluid around the developing larvae of the causative agents of lymphatic filariasis of humans (elephantiasis) also has an As-p18-like protein (Michalski et al. 2002). As a further approach to understanding the functions of this and other nemFABPs, structural studies of As-p18 were carried out on a ^15^N, ^13^C isotopically-labelled sample using solution NMR techniques. Here we report complete sequence-specific resonance assignments for As-p18 complexed with oleic acid.

## Methods and experiments

Recombinant As-p18 (Mei et al. 1997) was expressed in BL21 (λDE3) *Escherichia coli* cells using [^13^C,^15^N]-labelled M9 minimal medium. The His-tagged fusion protein was purified by nickel chelate chromatography, followed by a gel filtration chromatography as a polishing step. The removal of contaminating ligands derived from the bacterial expression system was achieved by reverse-phase (RP) chromatography with a C8 stationary phase and water/acetonitrile/trifluoroacetic acid mobile phase, followed by refolding in aqueous buffer. The protein was concentrated to approximately 0.5 mM in 20 mM sodium phosphate pH 7.20, and D_2_O was added to a final concentration of 5 % (v/v) prior to data acquisition. The protein was saturated with oleic acid by adding a small volume of a concentrated solution of oleic acid in deuterated ethanol. All spectra were recorded at 298 K on a Bruker Avance spectrometer at 600 MHz equipped with a TCI cryoprobe. Proton chemical shifts were referenced relative to the H_2_O offset frequency and heteronuclear chemical shifts calculated from the proton reference (Wishart et al. 1995).

Sequence-specific resonance assignment of the As-p18 backbone was accomplished with the aid of 2D ^15^N-HSQC, 3D HNCACB, 3D CBCA(CO)NH (Muhandiram and Kay 1994) 3D HNCO (Kay et al. 1994) and 3D HNCACO spectra. The majority of aliphatic sidechain carbon and proton resonances were located by navigating from the backbone data using 2D ^13^C-HSQC, 3D (H)C(CO)NH-TOCSY, 3D HBHA(CBCA)NH (Wang et al. 1994) and 3D H(C)(CO)NH-TOCSY spectra (Grzesiek and Bax 1992). Remaining aliphatic resonances were identified using 3D HCCH-TOCSY (Kay et al., 1993) or 3D ^13^C-edited [^1^H, ^1^H]-NOESY spectra. A proportion of aromatic sidechain ^13^C/^1^H signals (histidine H_δ1_, tryptophan H_δ1_, tyrosine H_δ,ε_ and phenylalanine H_δ,ε_) were assigned using 2D HBCBCGCDHD and 2D HBCBCGCDCEHE spectra (Yamazaki et al. 1993) and the remainder were identified from the ^13^C-edited [^1^H, ^1^H]-NOESY spectrum. Methionine C_ε_/H_ε_ were identified with the help of multiplicity edited CT-HSQC (Vuister and Bax 1992). NMR spectra were processed using AZARA (http://www.bio.cam.ac.uk/azara) and analysed with CCPNmr analysis (Vranken et al. 2005).

## Extent of assignments and data deposition

**Fig. 1:**
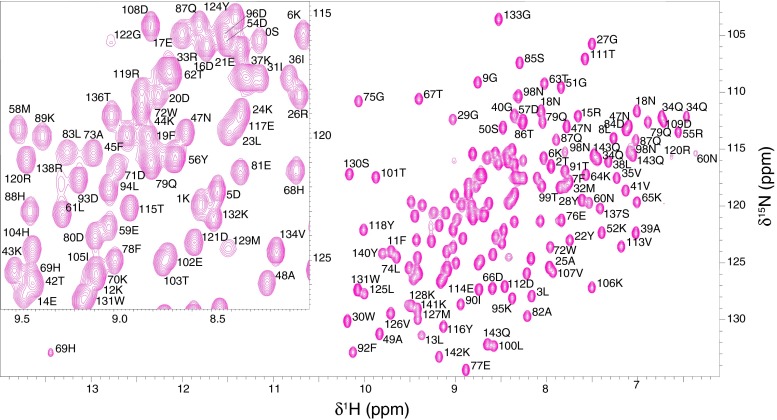
Two-dimensional ^1^H -^15^N HSQC spectrum of recombinant As-p18. All assigned crosspeaks have been labelled with the single letter amino acid code and their position in the amino acid sequence. The *inset* shows an expanded view of the region between ^1^H; 8.00–9.58 and ^15^N; 114.40–127.00 ppm of the HSQC spectrum

All As-p18 polypeptide backbone resonances were assigned, with the exception of the disordered N-terminal His-tag residues which are too overlapped to resolve and whose backbone amide resonances are not observed. The amide resonance of Q18, W30, D123 and Y139 were not observed, probably due to rapid exchange with solvent water. Figure 1 shows the ^1^H-^15^N HSQC spectrum with the assignments indicated. Backbone resonance assignments for the native sequence^1^ (excluding the flexible His-tag) have been obtained for 97.1 % of the non-proline H_N_/N pairs (135 out of 139), 99.3 % of the H_α_ (150 out of 151), 97.9 % of the C_α_ (140 out of 143), and 99.3 % of the C_β_ (134 out of 135) atoms. In addition, assignment for 99.2 % of the H_β_ and >93 % of the H_γ_ and H_δ_ resonances have been assigned. Most of the missing assignments along the residue sidechain are those of lysine C_ε_H_ε_ groups that are too overlapped in both ^1^H and ^13^C dimensions to be resolved. No resonances were observed for the following NH_n_ groups: arginine N_ε_H_ε_, N_η1_H_2_, N_η2_H_2_ (with the exception of R120 N_ε_H_ε_), histidine N_δ1_H_δ1_, N_ε1_H_ε1_ (with the notable exception of H69 N_δ1_H_δ1_ at 169.1/13.45 ppm) and lysine N_ζ3_H_3_.

**Fig. 2:**
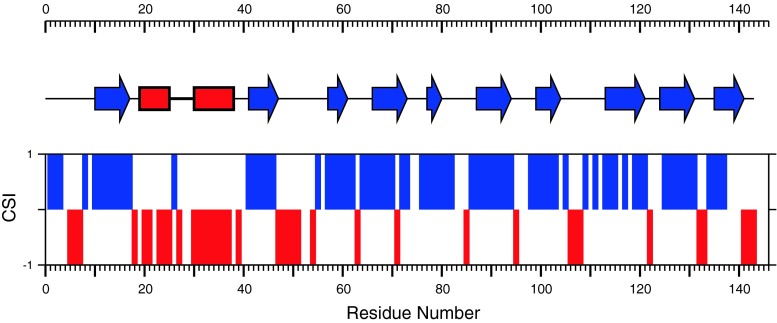
Alignment of CSI consensus values (*bars*) with the predicted secondary structure elements of As-p18. *Positive* and *negative CSI values* indicate β-strand and α-helix segments, respectively. The secondary structure prediction, was calculated using DANGLE for assigned Cα, Cβ, Hα, C’ and NH. *Blue arrows* and *red rectangles* represent β-strands and α-helical stretches, respectively

The ^13^C chemical-shift index (CSI; Wishart and Sykes 1994) and secondary structure analysis using DANGLE (Cheung et al. 2010) indicate the presence of two α-helices and ten β-strands (Fig. 2), and the arrangement of secondary structural elements as a function of sequence is similar to that discovered in FABPs whose structures have previously been determined (Banaszak et al. 1994). However, the chemical shift data predict an extended loop between strands βB and βC, spanning amino acids 47–55 which may be a feature of nemFABPs in general.

The ^1^H, ^13^C and ^15^N chemical shift assignments have been deposited with the BioMagResBank database (http://www.bmrb.wisc.edu), accession number 18632.

## Footnotes

1 Native sequence spans residues K1-Q143. Negative numbering accounts for the twelve residues in the His-tag at the N-terminus of recombinant As-p18.
